# Cortisol and development of depression in adolescence and young adulthood – a systematic review and meta-analysis

**DOI:** 10.1016/j.psyneuen.2021.105625

**Published:** 2022-02

**Authors:** Zuzanna Zajkowska, Nancy Gullett, Annabel Walsh, Valentina Zonca, Gloria A. Pedersen, Laila Souza, Christian Kieling, Helen L. Fisher, Brandon A. Kohrt, Valeria Mondelli

**Affiliations:** a King’s College London, Department of Psychological Medicine, Institute of Psychiatry, Psychology & Neuroscience, London, UK; b Biological Psychiatry Unit, IRCCS Istituto Centro San Giovanni di Dio Fatebenefratelli, Brescia, Italy; c Division of Global Mental Health, Department of Psychiatry, School of Medicine and Health Sciences, The George Washington University, 2120L St NW, Ste 600, Washington, DC 20037, USA; d Department of Psychiatry, Universidade Federal do Rio Grande do Sul, Child & Adolescent Psychiatry Division, Hospital de Clínicas de Porto Alegre, Rua Ramiro Barcelos, 2350 – 400N, Porto Alegre, RS 90035–903, Brazil; e King’s College London, Social, Genetic & Developmental Psychiatry Centre, Institute of Psychiatry, Psychology & Neuroscience, London, UK; f ESRC Centre for Society and Mental Health, King’s College London, London, UK; g National Institute for Health Research (NIHR) Maudsley Biomedical Research Centre, South London and Maudsley NHS Foundation Trust, King's College London, London, UK

**Keywords:** Depression, Adolescence, Cortisol, HPA axis, Stress, Major depressive disorder

## Abstract

**Introduction:**

Dysregulation of the hypothalamic-pituitary-adrenal (HPA) axis has been implicated in the development of major depressive disorder (MDD) in adulthood. Less work has focused on the role of the HPA axis in depression in adolescence and young adulthood globally. The aim of this study was to conduct a systematic review and meta-analysis of worldwide research investigating the relationship between cortisol, a measure of HPA axis activity, and MDD in adolescence and young adulthood.

**Method:**

We searched MEDLINE, PsycINFO, Cochrane Database of Systematic Reviews, Web of Science, Lilacs, African Journals Online, and Global Health for studies which examined the relationship between cortisol and MDD in global youth (10–24 years old).

**Results:**

Twenty-six studies were included in the systematic review and 14 were eligible for the meta-analysis, but only one study included young adults in their sample. Results from the meta-analysis demonstrated that elevated morning, but not evening, cortisol levels was prospectively associated with later MDD development in adolescence and young adulthood. However, morning cortisol levels did not significantly differ between healthy controls and individuals with MDD in cross-sectional studies. Afternoon cortisol and cortisol stress response also did not differ between adolescents with MDD and healthy controls. Qualitative synthesis of the three studies examining nocturnal cortisol showed higher nocturnal cortisol was both longitudinally and cross-sectionally associated with MDD in adolescence.

**Conclusion:**

Our findings suggest elevated morning cortisol precedes depression in adolescence. Despite this, we did not find any differences in other cortisol measures in association with MDD in cross-sectional studies. Taken together, these findings suggest that elevated morning and nocturnal cortisol are risk factors for depression in adolescence rather than a biomarker of existing MDD. This supports a role for the hyperactivity of the HPA axis in the development of MDD in adolescence. Most of the studies were from high-income-countries (HICs) and thus further work would need to be conducted in low- and middle-income countries (LMICs) to understand if our findings are generalisable also to these populations.

## Highlights

• Higher morning cortisol is associated with later MDD development in adolescence.
• Morning cortisol is not associated with adolescent MDD in cross-sectional studies.
• Morning and nocturnal cortisol are risk factors for depression in adolescence.

## 1 Introduction

Given the high incidence of depression in the first decades of life and chronicity throughout the life course, adolescence and young adulthood represent a window of opportunity to develop effective prevention strategies and reduce the burden of disease. Understanding the biological mechanisms involved in the development of depression in youth is an important step to develop such strategies (Whiteford et al., 2013). A compelling body of evidence implicates dysregulation of the hypothalamic-pituitary-adrenal (HPA) axis in the pathophysiology of MDD in adulthood (Kennis et al., 2020, Stetler and Miller, 2011). Evidence from cross-sectional studies has repeatedly shown higher levels of cortisol in those with MDD compared with healthy controls (Islam et al., 2018, Khan et al., 2019). Elevated cortisol levels have also been found to prospectively predict subsequent onset of MDD (Vrshek-Schallhorn et al., 2013), including findings from the recent meta-analysis by Kennis and colleagues (Kennis et al., 2020). However, the majority of studies examining cortisol as a biological risk factor for depression focus on adults (Kennis et al., 2020, Belvederi Murri et al., 2014, Stetler and Miller, 2011, Zorn et al., 2017). Although there is a growing body of research suggesting that similar mechanisms are involved in child and adolescent populations (Lopez-Duran et al., 2009), there is still little known about the role of cortisol in MDD onset in adolescents and young people. There are several pathways through which the HPA axis dysregulation may be involved in the aetiology of depression. For example, glucocorticoid resistance, whereby the glucocorticoid receptor-mediated negative feedback becomes impaired, is one of the main theories put forward for the HPA axis dysregulation and increased levels of cortisol in patients with depression (Cattaneo et al., 2020, Nikkheslat et al., 2020). Cortisol, the main HPA axis hormone and the key glucocorticoid produced in response to stress, regulates neuronal survival and neurogenesis. One hypothesis suggests that high levels of circulating cortisol can lead to a reduction in neurogenesis which in turn can contribute towards symptoms of depression (Anacker, 2014). Furthermore, the hyperactivation of the HPA axis leads to an increase in circulating inflammatory cytokines, which have been widely implicated in the pathogenesis of depression (Dowlati et al., 2010, Hiles et al., 2012, Liu et al., 2012, Nettis et al., 2021, Nikkheslat et al., 2020). Increased inflammatory cytokines are hypothesised to contribute towards the pathogenesis of depression in several ways. For example, they activate enzyme Indoleamine-2–3-Dioxygenase (IDO) which, through the kynurenine pathway, leads to a reduction in serotonin (Miller and Raison, 2016), a key neurotransmitter known to be involved in depression (Kambeitz and Howes, 2015). Increased levels of inflammatory cytokines are also suggested to reduce levels of an important neurotrophic factor, Brain Derived Neurotrophic Factor (BDNF), which is hypothesised to be another key mechanism underlying development of depression (Lee and Kim, 2010). Lastly, an activation of the immune system can contribute to brain related abnormalities including structural and functional changes which have been reported in depression (Opel et al., 2019).

Whilst there have been several meta-analyses investigating cortisol in depression in adults (Broderick et al., 1998, Knorr et al., 2010, Stetler and Miller, 2011), only two were conducted including adolescent populations. Lopez and colleagues (Lopez-Duran et al., 2009) showed higher cortisol levels in children and adolescents with depression compared with healthy volunteers. Kennis and colleagues (Kennis et al., 2020) showed that higher cortisol predicted subsequent MDD onset in a sample including both adolescents and adults, the effect which was no longer present when patients with MDD at baseline were excluded. However, none of the meta-analyses focused on the period of adolescence and young adulthood, which is an important period of growth and transition from childhood to adulthood, where biological maturational changes take place (Miller et al., 2015). The increase in suicide rates from adolescence to young adulthood and, over the past fifty years, a smaller decrease in mortality rates among young people compared with other age groups, are clear examples of why focusing on this age group is very important in informing effective prevention strategies in youth depression (World Health Organisation, 2011). Focusing on the age span of adolescence and young adulthood also maximises the likelihood of capturing first onset depression and the risk factors associated with it. Given the maturational changes of prefrontal cortex continue into early 20’s, and changes in social roles extend beyond the age of 19, capturing the neurobiological changes during this transition period is key to understand the wider picture of the mechanisms underpinning depression development during this transition period from childhood to adulthood (Miller et al., 2015, Sawyer et al., 2018). Lastly, previous meta-analyses did not use extensive range of electronic databases to include research conducted in low-and-middle-income-countries (LMICs).

Therefore, to address these gaps, the aim of this study was to conduct a systematic review and meta-analysis of worldwide-based studies, using an extensive range of electronic databases, looking at different types of cortisol measures as a risk factor for MDD development and presence in adolescents and young adults (age 10–24), as defined by the World Health Organisation (World Health Organisation, 2011), including longitudinal and cross-sectional studies, and intervention and prevention trials.

## 2 Methods

### 2.1 Literature search strategy

The systematic review and meta-analysis were conducted in accordance with the Preferred Reporting Items for Systematic Reviews and Meta-Analyses (PRISMA) (Moher et al., 2015). We searched the following electronic databases since inception until 24th July 2020: MEDLINE (via Ovid), PsycINFO, Cochrane Database of Systematic Reviews, Web of Science (Core Collection), Lilacs, African Journals Online, and Global Health. We conducted searches in English, however publication language was not a restriction. Only published research in academic journals was eligible for inclusion. We performed searches with the following terms: adolesc* OR young adult OR youth OR teen* OR young people OR young person* AND depression OR depressive disorder OR symptoms of depression OR major depressive disorder AND HPA axis OR pituitary OR cortisol OR adrenal OR glucocorticoid* . In addition to electronic searches, we hand-searched relevant systematic reviews and reference lists of the retrieved articles for eligible studies that may have been missed.

### 2.2 Eligibility of the studies

The studies were included if they met the following inclusion criteria: 1) presence of MDD through a categorical diagnostic interview or continuous measure of depressive symptoms such as a questionnaire with the cut-off value for clinical symptoms of depression reported; 2) adolescents and young people age range 10–24; 3) measurement of cortisol as a risk factor for developing MDD in adolescence; 4) design: any intervention or prevention trial, cross-sectional or longitudinal study; 5) articles published in peer reviewed journals globally. Studies were excluded if they met at least one of the following criteria: 1) individuals included were limited to only specific medical subpopulations, e.g., HIV, diabetes, intellectual disabilities etc; 2) non-research papers; 3) qualitative studies; 4) no diagnosis of depression; 5) depressive symptoms measured without a cut-off value representing clinical symptoms of depression; 6) reported follow-up period of < 6 months (for longitudinal studies).

### 2.3 Analysis

Full text screenings and quality assessments were conducted by four independent reviewers (NG, AW, VZ, ZZ) until inter-rater reliability of > 90% agreement was reached, following which assessments of articles for eligibility were performed independently. Separate meta-analyses were conducted split by type of cortisol measure used (for example, morning cortisol, afternoon cortisol and cortisol stress response). As times of cortisol collection differed across studies, to keep consistency, we applied the following rules: a) morning cortisol included samples collected in the morning, where possible within the first hour of awakening, b) afternoon cortisol included any samples taken between 12 and 6 pm, c) and evening cortisol – samples collected after 6 pm until night-time. In regard to morning cortisol, when variation in cortisol levels changes rapidly in the first hour from awakening, where multiple time points of collection were reported, we opted for samples collected at least 30 min after awakening. Three studies reported specific times from awakening which were 50, 40 and 30 min, respectively (Adam et al., 2010, Carnegie et al., 2014, Goodyer et al., 2010). The remaining studies reported only time of collection and these were as follows: 8 am (Goodyer et al., 2003, Goodyer et al., 2000a, Goodyer et al., 2000b, Halligan et al., 2007, Owens et al., 2014), between 7:30 and 8:30 am (Dorn et al., 1996), between 9 and 10 am (Birmaher et al., 1994), between 8 and 9 am (De Villiers et al., 1989) and upon waking (Tonon et al., 2020). Given the small number of studies available within each type of cortisol measure category, we performed meta-analyses where there was a minimum of two studies which investigated cortisol as a risk factor for depression using comparable methods. We extracted means and SDs of MDD and healthy controls (HC) and performed random effects model using standardized mean differences (SMDs), and 95% confidence intervals (CI). Where means and SDs were not reported in the article, we contacted authors of the article to request the relevant data. We assessed heterogeneity between the studies using I2 statistic with the thresholds of 25%, 50% and 75% for low, moderate and high heterogeneity, respectively. The analysis was performed using Cochrane Review Manager version 5.3.24. We used SAQOR quality assessment criteria with modified GRADE rankings (see Table 2). Twenty-two studies were marked as “adequate” across four or more of the six SAQOR categories and remained at the GRADE rating for observational studies of “low” quality. The remaining studies (n = 4) were downgraded to a GRADE rating of “very low”. The final quality modified GRADE rating reported in our review was based on the study design and the number of SAQOR categories marked as “adequate”. All of the studies reported were observational. An observational study with a minimum of four “adequate” categories as per SAQOR, was graded as “low”. A study having less than four categories marked as “adequate” meant that the study was graded as “very low” according to the modified GRADE rating. Therefore, the maximum GRADE rating for these studies was 'low' given that they all used observational designs (see Table 1). We used the World Bank Country classification system to determine most recent economy classification for each country (The World Bank, 2021).

**Table 1:** Description of included studies examining the relationship between cortisol levels and MDD in adolescence and young adulthood (n = 26).

First author (year)	Type of cortisol sample	Study design	Sample size				Outcome measure#	Study setting (HIC or LMIC)^
			LR*	HR**	MDD***	HC****		
Tonon (2020)	Salivary cortisol - morning, afternoon, evening and night	Cross-sectional	··	··	44	192	BDI-II (a score of 11 or higher on the BDI-II was used to identify clinically significant depressive symptoms)	LMIC
Ming (2017)	Salivary cortisol – cortisol levels in response to psychosocial stress	Cross-sectional	··	··	36	36	SCID	LMIC
Morris (2017)	Salivary cortisol - cortisol levels in response to psychosocial stress	Cross-sectional	72^a^	35	38	··	K-SADS, CDRS-R	HIC
Shenk (2015)	Salivary cortisol – cortisol levels in response to psychosocial stress	Longitudinal	··	··	MDD with child maltreatment n = 51, MDD without child maltreatment n = 59	··	BDI-II (a score of 21 or higher on the BDI-II was used identify clinical levels of MDD symptoms)	HIC
Grant (2015)	Salivary cortisol - morning and evening cortisol ratio	Cross-sectional	··	··	Mild depression n = 46, Moderate depression n = 48, Severe depression n = 18, Extremely severe depression n = 18	314	DASS	HIC
Carnegie (2014)	Salivary cortisol –morning cortisol, cortisol awakening response (CAR) and afternoon cortisol	Longitudinal (for meta-analysis, cross-sectional baseline data available)	··	··	MDD at follow up n = 46	HC at follow-up n = 622	CIS-R	HIC
Klimes-Dougan (2014)	Salivary cortisol – cortisol levels in response to psychosocial stress	Cross-sectional	··	··	52	27	K-SADS, CDRS, BDI	HIC
Owens (2014)	Salivary cortisol – morning cortisol	Longitudinal (for meta-analysis cross-sectional data from the two cohorts combined used)	··	660	MMD at follow up n = 770	··	K-SADS, MFQ	HIC
Morris (2014)	Salivary cortisol – cortisol levels in response to psychosocial stress	Cross-sectional	··	··	24	26	K-SADS, BDI; LIFE	HIC
Vrshek-Schallhorn (2013)	Salivary cortisol – cortisol awakening response (CAR), the diurnal rhythm slope and the average cortisol level	Longitudinal	··	270	MDD at follow up n = 42	··	SCID	HIC
Ulrike (2013)	Salivary cortisol – cortisol awakening response (CAR)	Cross-sectional	··	··	63	68	Kinder-DIPS von	HIC
Adam (2010)	Salivary cortisol - wake-up, wake-up plus 40 min values, and bedtime values, size of the cortisol awakening response (CAR), slope of the diurnal cortisol rhythm from wake-up to bedtime, evening cortisol average cortisol	Longitudinal	··	230	MMD AT follow-up n = 19	··	SCID	HIC
Goodyer (2010)	Salivary cortisol – morning cortisol	Longitudinal	··	401	MDD at follow-up. n = 41	··	K-SADS, MFQ	HIC
Rao (2009b)	Nocturnal urinary free cortisol (NUFC)	Longitudinal	··	48	MDD at follow-up n = 14	48	K-SADS; LIFE	HIC
Rao (2009a)	Nocturnal urinary free cortisol (NUFC)	The study design is longitudinal however, the analysis extracted in this review, looking at cortisol and depression, is cross-sectional	··	··	55	48	K-SADS, HDRS, BDI	HIC
Rao & Poland (2008b)	Nocturnal urinary free cortisol (NUFC)	Cross-sectional	··	··	16	16	K-SADS-PL	HIC
Rao et al. (2008a)	Salivary cortisol – cortisol in response to psychosocial stressor	Cross-sectional	··	··	30	25	K-SADS, HDRS, BDI	HIC
Halligan (2007)	Salivary cortisol – morning and evening cortisol	Longitudinal	··	39	MDD at follow-up n = 25	39	MFQ (with cut-off score of 7 and above)	HIC
Goodyer (2003)	Salivary cortisol – morning and evening cortisol	Longitudinal	··	60		··	K-SADS	HIC
Goodyer (2000b)	Salivary cortisol – morning and evening cortisol	Longitudinal	··	180	MDD at follow up n = 48	··	K-SADS	HIC
Goodyer (2000a)	Salivary cortisol – morning and evening cortisol	Longitudinal	65^b^	181	MDD at follow=up n = 31	··	K-SADS	HIC
Ghaziuddin (2000)	Plasma - cortisol response to *meta*-chlorophenylpiperazine (*m*CPP*)* infusion (a serotonin agonist)	Cross-sectional	··	··	12	12	DISC	HIC
Dorn (1996)	Serum – morning cortisol, cortisol response to ovine corticotrophin releasing hormone (oCRH) test before and after a cognitive stressor Urinary free cortisol (UFC) – 24 h.	Cross-sectional	··	··	21	20	DISC	HIC
Birmaher (1994)	Plasma - morning cortisol	Cross-sectional	··	··	20	20	K-SADS	HIC
Kutcher (1991)	Serum – nocturnal cortisol	Cross-sectional	··	··	12	HC sample size not reported	K-SADS	HIC
De Villiers (1989)	Plasma – morning cortisol	Cross-sectional	··	··	10	17	ISC	LMIC

* Low-risk for depression – ^a^ adolescents with no personal or family history of a psychiatric disorder, ^b^ 1) no moderate or severe life events in last 12 months, 2) no current marital disharmony or past marital breakdown, 3) no lifetime exit events (bereavement and/or permanent separation) of personal significance to the adolescent (i.e. involving a relative or friend), 4) high (>80th percentile) emotionality.

** High-risk for depression – definitions vary by articles, please refer to individual articles for specific definitions.

*** Major depressive disorder

**** Healthy-control

**#**Outcome measure abbreviations: Beck Depression Inventory (BDI); Depression, Anxiety, Stress scales (DASS) questionnaire; The Clinical Interview Schedule-Revised (CIS-R); Kiddie-Schedule for Affective Disorders and Schizophrenia (K-SADS); Diagnostic Interview Schedule for Children (DISC); Structured Clinical; Interview for Diagnostic and Statistical Manual-IV (SCID); Moods and Feelings Questionnaire (MFQ); Depression Scale for Children (DSC); Longitudinal Interval Follow-up Evaluation (LIFE); The Diagnostisches Interview bei psychischen Sto¨rungen im Kindesund Jugendalter (Kinder-DIPS von); Children Depression Rating Scale (CDRS); Hamilton Rating Scale for Depression (HRSD); Hamilton Depression Rating Scale (HDRS); Epidemiologic Studies Depression Scale (CES-D); Interview Schedule for Children (ISC)

^High-income countries (HICs); Low-and-middle-income countries (LMICs)

··N/A

**Table 2:** Systematic Assessment of Quality in Observational Research (SAQOR) Quality Assessment ratings of included studies (n = 26).

First author (year)	Sample	Control group/comparison group	Measurement quality	Follow-up	Distorting Influences	Reporting of data	Modified Grade Rating
Adam et al. (2010)	Adequate	Inadequate	Adequate	Inadequate	Adequate	Adequate	Low
Birmaher et al. (1994)	Adequate	Adequate	Adequate	··	Adequate	Adequate	Low
De Villiers et al. (1989)	Adequate	Adequate	Adequate	··	Inadequate	Inadequate	Very low
Dorn et al. (1996)	Adequate	Adequate	Adequate	··	Adequate	Adequate	Low
Carnegie et al. (2014)	Adequate	Inadequate	Adequate	Adequate	Inadequate	Adequate	Low
Ghaziuddin et al. (2000)	Adequate	Adequate	Adequate	··	Adequate	Adequate	Low
Goodyer et al. (2000a)	Adequate	Adequate	Adequate	Adequate	Adequate	Adequate	Low
Goodyer et al. (2000b)	Adequate	Adequate	Adequate	Adequate	Adequate	Inadequate	Low
Goodyer et al. (2003)	Adequate	Inadequate	Adequate	Adequate	Adequate	Inadequate	Low
Goodyer et al. (2010)	Adequate	Inadequate	Adequate	Adequate	Inadequate	Adequate	Low
Grant et al. (2015)	Adequate	Inadequate	Adequate	··	Inadequate	Adequate	Very low
Halligan et al. (2007)	Adequate	Adequate	Adequate	Adequate	Inadequate	Adequate	Low
Klimes-Dougan et al. (2014)	Adequate	Adequate	Adequate	··	Adequate	Adequate	Low
Kutcher et al. (1991)	Adequate	Adequate	Adequate	··	Adequate	Adequate	Low
Ming et al. (2017)	Adequate	Adequate	Adequate	Adequate	Adequate	Adequate	Low
Morris and Rao (2014)	Inadequate	Adequate	Adequate	··	Adequate	Adequate	Low
Morris et al. (2017)	Adequate	Adequate	Adequate	Inadequate	Adequate	Adequate	Low
Owens et al. (2014)	Adequate	Inadequate	Adequate	Adequate	Inadequate	Inadequate	Very low
Rao et al. (2008)	Inadequate	Adequate	Adequate	··	Adequate	Adequate	Low
Rao & Poland (2008)	Adequate	Adequate	Adequate	Adequate	Adequate	Adequate	Low
Rao et al. (2009a)	Adequate	Adequate	Adequate	Adequate	Adequate	Adequate	Low
Rao et al. (2009b)	Inadequate	Adequate	Adequate	Adequate	Adequate	Inadequate	Low
Shenk et al. (2015)	Adequate	Adequate	Adequate	Adequate	Adequate	Adequate	Low
Tonon et al. (2020)	Inadequate	Adequate	Adequate	Adequate	Adequate	Adequate	Low
Vrshek-Schallhorn et al. (2013)	Adequate	Inadequate	Adequate	Adequate	Adequate	Adequate	Low
Ulrike et al. (2013)	Adequate	Adequate	Inadequate	··	Adequate	Inadequate	Very low

··N/A

**Fig. 1:**
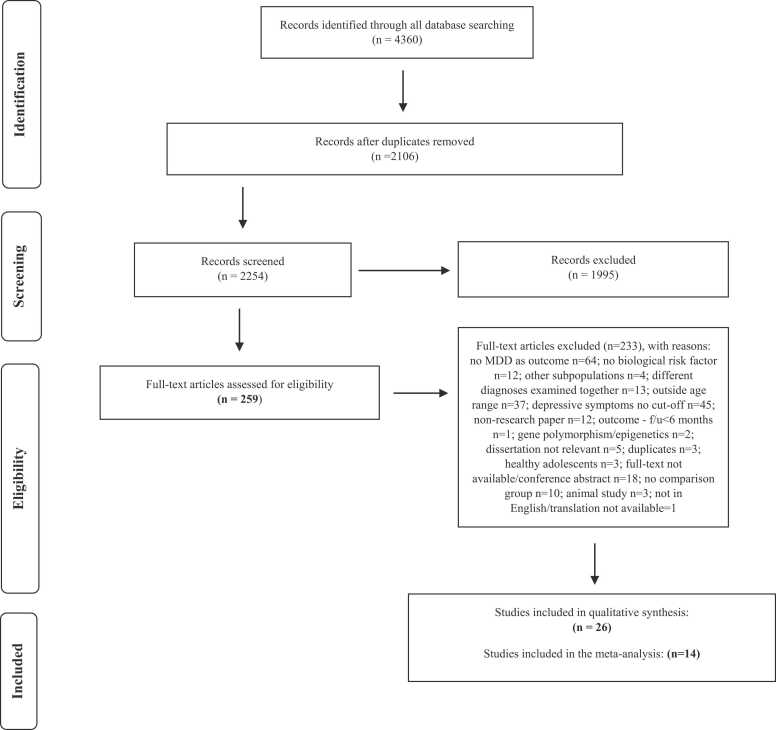
PRISMA Flow Diagram of Study Selection Process.

Twenty-six studies were considered eligible for the qualitative synthesis and 14 studies for meta-analysis (see Fig. 1 for the PRISMA flow diagram of the study selection process). Eligible articles included 14 cross-sectional studies and 12 longitudinal studies which we grouped based on study design and/or the type of cortisol measurement used, and onset of depression, and were as follows: 1) morning cortisol assessed longitudinally (8 studies), 2) morning cortisol assessed cross sectionally (6 studies), 3) cortisol awakening response (CAR) (4 studies), 4) diurnal rhythm slope (2 studies), 5) afternoon cortisol (2 studies), 6) evening cortisol (6 studies), 7) nocturnal cortisol (4 studies), 8) cortisol in response to a psychosocial stressor (7 studies), and 9) onset of depression (12 studies). Some studies overlapped as included different measures of cortisol (e.g., morning and afternoon cortisol) and/or both cross-sectional and longitudinal analyses. Three studies were conducted in LMICs (De Villiers et al., 1989, Ming et al., 2017, Tonon et al., 2020). All studies focused on adolescence and one included both adolescents and young people (Ming et al., 2017).

For the meta-analysis, we grouped the studies according to the type of cortisol measure used and performed five meta-analyses as follows: morning cortisol assessed longitudinally (n = 5); morning cortisol assessed cross sectionally (n = 6); afternoon cortisol (n = 2); evening cortisol (n = 3); cortisol stress response (n = 3). Three of the studies included in the meta-analysis of longitudinal morning cortisol overlap with the three articles analysed in the evening cortisol subgroup. In addition, two of the studies included in the meta-analysis of the cross-sectional morning cortisol overlap with the two articles analysed in the afternoon cortisol subgroup. We were unable to perform sensitivity analysis due to the small sample size for the cortisol subgroups and therefore cannot determine a clear source for heterogeneity. Characteristics of the studies are summarised in Table 1.

## 3 Results

### 3.1 Morning cortisol longitudinally assessed

Out of the eight studies that assessed morning cortisol longitudinally, four showed that adolescents who had higher morning cortisol levels at baseline were significantly more likely to develop MDD at a later time point (follow-up times ranged from 12 to 36 months) compared with those with lower baseline morning cortisol levels (Goodyer et al., 2010, Goodyer et al., 2000b, Halligan et al., 2007, Owens et al., 2014). The remaining four studies did not find an association between morning cortisol and subsequent development of MDD in adolescence (follow up times ranged from 12 to 36 months) (Adam et al., 2010, Carnegie et al., 2014, Goodyer et al., 2003, Goodyer et al., 2000a).

**Fig. 2:**
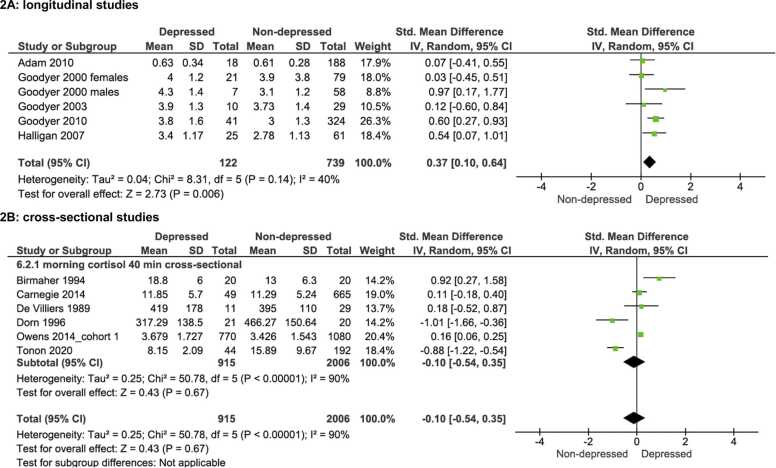
Forest plots of morning cortisol and adolescent depression in longitudinal studies (Fig. 2A) and in cross-sectional studies (2B).

Due to data not available or samples overlapping, we were able to run the meta-analyses looking at morning cortisol longitudinally on five studies out of eight, one of which looked at cortisol measures in male and females separately (Adam et al., 2010, Goodyer et al., 2010, Goodyer et al., 2003, Goodyer et al., 2000b, Halligan et al., 2007). A significant overall effect was found showing that higher baseline cortisol levels in adolescence were associated with the development of depression at a later stage (MDD n = 122; no MDD n = 739), (SMD= 0.37, 95% CI 0.10, 0.64, *p* = .006). The heterogeneity between the studies was low (χ^2^ = 8.31, I^2^ = 40%, *p* = .14) (see Fig. 2A).

### 3.2 Morning cortisol cross-sectionally assessed

In total six studies examined morning cortisol cross-sectionally by comparing morning cortisol levels in those with MDD and healthy controls. Evidence for an association between morning cortisol and depression was mixed, with two studies showing that adolescents with MDD had significantly higher levels of morning cortisol compared with healthy controls (Birmaher et al., 1994, Owens et al., 2014), whilst two other studies found that adolescents with MDD had significantly lower levels of morning cortisol compared with healthy controls (Dorn et al., 1996, Tonon et al., 2020). The remaining two studies found no significant difference in morning cortisol levels when measured either in plasma (De Villiers et al., 1989) or salivary cortisol (Carnegie et al., 2014) in adolescence with MDD compared with healthy controls.

In contrast to the findings looking at morning cortisol longitudinally, the meta-analysis including studies which assessed morning cortisol cross-sectionally (Birmaher et al., 1994, Carnegie et al., 2014, De Villiers et al., 1989, Dorn et al., 1996, Owens et al., 2014, Tonon et al., 2020) did not find a significant difference in morning cortisol levels in adolescents with a diagnosis of MDD (n = 915) compared with healthy controls (n = 2006), (SMD= −0.10, 95% CI −0.54, 0.35, *p* = .67). The heterogeneity between the studies was high (χ^2^ = 50.78, I^2^ = 90%, *p* < .001) (see Fig. 2B).

### 3.3 Cortisol awakening response (CAR)

One study reported that females with MDD had a significantly higher CAR compared with healthy control females (Ulrike et al., 2013). Furthermore, elevated CAR predicted MDD development one year and two and a half years later (Adam et al., 2010, Vrshek-Schallhorn et al., 2013). In contrast, one other study found no evidence that CAR was associated with the subsequent development of depression (Carnegie et al., 2014).

Although there were four studies investigating CAR in association with depression in adolescents, the heterogeneity between the methods of CAR measurement meant that it was not possible to compare these findings using meta-analysis.

### 3.4 Diurnal cortisol slope

Two studies assessed whether diurnal cortisol rhythm measured in salivary cortisol was associated with subsequent development of MDD at one-year follow-up (Adam et al., 2010) and over a four-year follow-up period (Vrshek-Schallhorn et al., 2013). Neither study found that diurnal rhythm was associated with subsequent development of depression.

### 3.5 Afternoon cortisol

**Fig. 3:**
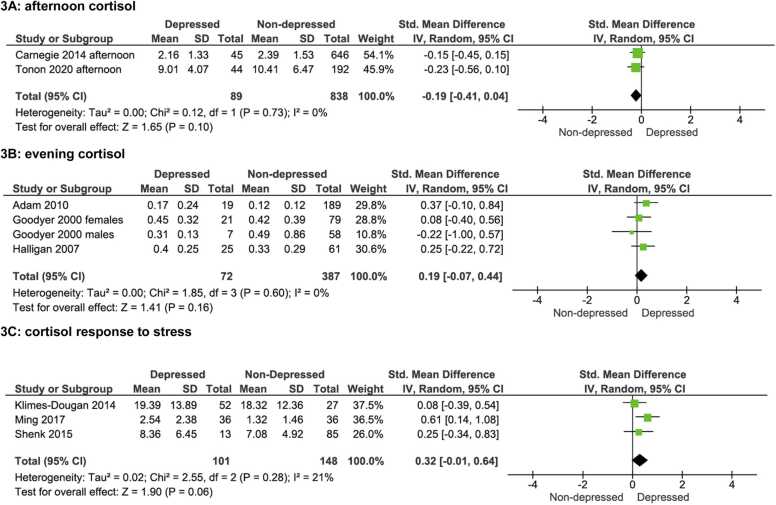
Forest plots of afternoon cortisol (3A), evening cortisol (3B), and cortisol response to stress (3C) in adolescents with MDD compared with healthy adolescents.

Two studies examined salivary cortisol measured in the afternoon in adolescents with depression and healthy controls and found no association between MDD diagnosis and afternoon cortisol levels (Carnegie et al., 2014, Tonon et al., 2020). The meta-analysis of these studies found no significant difference between the two groups (MDD: n = 89, no MDD: n = 838; SMD = −0.19, 95% CI −0.41, 0.04, *p* = .10). The heterogeneity between the studies was low (χ^2^ = 0.12, I^2^ = 0%, *p* = .07) (see Fig. 3A).

### 3.6 Evening cortisol

Out of the six studies which examined evening cortisol, five of the studies were longitudinal, examining evening cortisol in relation to the subsequent development of MDD 12, 24 or 36 months later (Adam et al., 2010, Goodyer et al., 2003, Goodyer et al., 2000a, Goodyer et al., 2000b, Halligan et al., 2007). None of these studies found that evening cortisol levels were associated with subsequent development of MDD at the follow-up. The one remaining study compared evening cortisol in those with MDD with healthy controls cross-sectionally and found no significant difference between the groups (Tonon et al., 2020). Due to samples overlap and data not available, we ran a meta-analysis on three of the longitudinal studies (Adam et al., 2010, Goodyer et al., 2000a, Halligan et al., 2007), one of which was analysed in males and females separately. No overall significant difference was found between baseline evening cortisol levels and MDD development at the follow-up (MDD: n = 72; no MDD: n = 387), (SMD= 0.19, 95% CI −0.07, 0.44, *p* = .16). The heterogeneity between the studies was low (χ^2^ = 1.85, I^2^ = 0%, *p* = .60) (see Fig. 3B).

### 3.7 Nocturnal cortisol

Rao and colleagues reported elevated nocturnal urinary free cortisol (UFC) in adolescents at high-risk for MDD, by virtue of parental depression, who developed depression compared with those who did not (Rao et al., 2009b). The same group also reported in two other studies that adolescents with MDD had significantly higher levels of nocturnal UFC compared with healthy controls (Rao et al., 2009a, Rao and Poland, 2008). In contrast, one other study which measured nocturnal cortisol via blood serum found no significant difference between cortisol levels in those with MDD compared with healthy controls (Kutcher et al., 1991).

### 3.8 Cortisol stress response

Two studies reported that youth with MDD showed greater cortisol response to social stress tests compared with healthy controls (Ming et al., 2017, Rao et al., 2008). Morris and colleagues reported that adolescents with MDD did not show habituation of cortisol response to repeated stress exposure as reported in healthy adolescents (Morris and Rao, 2014). In contrast, a third study demonstrated that participants with MDD and those at high-risk by virtue of having one or both parents with MDD, reported significantly lower cortisol reactivity in response to the Trier Social Stress Test (TSST) compared with healthy controls (Morris et al., 2017). Furthermore, two additional studies found that there was no difference in cortisol levels in response to a psychosocial stressor between depressed adolescents and controls (Dorn et al., 1996, Klimes-Dougan et al., 2014). Finally, one other study tested whether cortisol had a specific indirect effect on the relationship between childhood maltreatment and the development of MDD and found that cortisol was not significantly associated with subsequent depression outcome (Shenk et al., 2015).

After conducting the meta-analysis of cortisol response to stress for these studies, (Klimes-Dougan et al., 2014, Ming et al., 2017, Shenk et al., 2015) adolescents with MDD (n = 101) had higher cortisol levels in response to stress compared with adolescents without depression (n = 148), but this did not reach statistical significance (SMD = 0.32, 95% CI −0.01, 0.64, p = .06). The heterogeneity between the studies was low (χ^2^ = 2.55, I^2^ = 21%, p = .28) (see Fig. 3C).

### 3.9 Cortisol and onset of depression

Out of twelve studies, two have shown that elevated CAR predicted subsequent depression onset regardless of whether it was a first or recurrent episode, however the prediction was stronger for recurrent depression (Adam et al., 2010, Vrshek-Schallhorn et al., 2013). Two more longitudinal studies have shown that elevated morning and nocturnal urinary free cortisol (NUFC) levels predicted first onset of MDD at follow-up, and one cross-sectional study reported higher NUFC levels in patients with MDD compared with healthy controls (Goodyer et al., 2010, Rao et al., 2009b, Rao and Poland, 2008). However, two other studies reported no association between morning and evening cortisol (first onset MDD), and CAR levels (first onset and recurrent depression) and subsequent MDD development (Carnegie et al., 2014, Goodyer et al., 2003). Furthermore, another study reported that cortisol levels in response to stress were higher in both adolescents with first episode and remitted depression compared with healthy controls (Ming et al., 2017). Three cross-sectional studies did not find any differences in morning, evening and nocturnal cortisol levels between adolescents with first episode depression and their healthy counterparts (De Villiers et al., 1989, Goodyer et al., 2000a, Kutcher et al., 1991). Lastly, no difference in CAR levels was reported in a cross-sectional study including adolescents with recurrent depression (Ulrike et al., 2013).

### 3.10 Other studies

Dorn and colleagues (Dorn et al., 1996) reported no significant difference in 24-hour urinary cortisol between adolescence with MDD and healthy controls. Grant and colleagues (Grant et al., 2015) looked at the ratio between waking and bedtime cortisol measures as an indicator of stress, where higher ratio represented lower state of stress as health cortisol secretion is expected to be high in the morning and low in the evening. They found that lower waking to bedtime cortisol ratio was predictive of mild depression in boys with a body mass index (BMI) of above 23.

One other study compared cortisol levels in response to meta-chlorophenylpiperazine (mCPP) infusion in patients with MDD and healthy controls (Ghaziuddin et al., 2000). Ghaziuddin and colleagues found that those in the MDD group showed a sharper baseline-cortisol decline between 08.00 am and 11.00 am and an increased response to the challenge compared with healthy controls.

## 4 Discussion

Our meta-analysis showed that morning, but not evening, cortisol was prospectively associated with later MDD development in adolescent populations. However, morning cortisol levels did not significantly differ between healthy controls and individuals with MDD in cross-sectional studies. Afternoon cortisol and cortisol stress response also did not differ between adolescents with MDD and healthy controls. Qualitative synthesis showed mixed results across different types of cortisol measure. Of note, two studies which reported lower cortisol levels in MDD compared with healthy controls included samples collected at earlier time-points, i.e., at waking and between 7:30 and 8 am, compared with the other studies looking at morning cortisol, where collection times ranged between 8 and 10 am. This highlights the importance of timing at which the cortisol samples are collected in the morning. Indeed, elevated CAR was shown to be associated with MDD in adolescents as reported in our qualitative analysis of this systematic review. Unfortunately, it was not possible to run a meta-analysis of the CAR data due to heterogeneity between the ways CAR was reported across the studies.

The main findings from our meta-analysis suggest that elevated morning cortisol precedes subsequent MDD onset in adolescence, regardless of being a first or recurrent episode of depression. This may suggest that elevated cortisol might be a predictor rather than a consequence of depression such that cumulative exposure to stress which initially results in elevated cortisol, with time, leads to blunted cortisol response which overlaps with MDD onset (Lam et al., 2019). This is an important finding because it suggests that the biological changes associated with transition to depression might be happening during the critical period of adolescence. Interestingly, previous meta-analysis, reported that children and adolescents with MDD showed higher levels of cortisol compared with healthy controls (Lopez-Duran et al., 2009). This suggests that initial elevation in cortisol levels, likely caused by the exposure to stress, is an antecedent of subsequent MDD development and that by the time one reaches adolescence, the cumulative stress exposure can lead to blunted cortisol levels and MDD development (Lam et al., 2019). Indeed, when looking at adolescent and adult populations together, Kennis and colleagues reported that elevated cortisol was associated with MDD onset at a later stage (Kennis et al., 2020). As such, elevated morning cortisol levels, as reported in our findings, may serve as a biomarker of risk for MDD onset in adolescence and our systematic review is the first to report it in adolescent populations.

Our findings further highlight the importance of longitudinal studies in adolescence, considering that this period and early adulthood constitute the peak for new cases, in many cross-sectional designs the individuals at-risk might be inadvertently classified as “healthy controls”, whilst some of them might develop depression which is not depicted. Therefore, a more comprehensive way of understanding depression and the risk factors associated with depression onset, is crucial to more accurately identify who is at-risk of developing this condition which would allow designing better prevention strategies (Kieling et al., 2021).

Whilst the only meta-analytic finding that reached significance was for morning cortisol assessed longitudinally, qualitative synthesis of associations between cortisol measured at different times of the day and depression showed that elevated CAR and nocturnal urinary free cortisol were associated with MDD, further supporting the hypothesis that HPA axis hyperactivation may be a risk factor for adolescent depression. Although not eligible for meta-analysis, all three studies which assessed nocturnal cortisol, support that elevated cortisol is associated with depression (Rao et al., 2009a, Rao et al., 2009b, Rao and Poland, 2008). Findings were from both cross-sectional and prospective longitudinal studies suggesting hyperactive HPA axis activity during the night may be both predictive and characteristic of adolescent depression. Furthermore, there is evidence for an association between higher cortisol awakening response (CAR) and depression. Two studies show that higher CAR is associated with later depression onset (Adam et al., 2010, Vrshek-Schallhorn et al., 2013) and one other study shows, those with MDD are characterised by elevated CAR (Ulrike et al., 2013). Our finding that hyperactivity of the HPA axis is associated with depression in adolescence, may suggest that high cortisol levels are a marker of high levels of stress that the adolescent is facing, and that may contribute to the development of depression at a later stage. They are also is in line with findings reported in adults which also suggest that hyperactive HPA axis activity, indicted by elevated cortisol levels (Pariante and Lightman, 2008, Varghese and Brown, 2001), is associated with MDD. Hyperactivity of the HPA axis observed in the studies reported in our review, indicate that glucocorticoid resistance may be present not only in adolescents with current MDD but also in those who are at-risk for developing depression and can act as a “switch” to the cascade of molecular mechanisms typically involved in depression, such as reduced neurogenesis, increased inflammation, and brain abnormalities (Opel et al., 2019). This is important in the context of designing prevention and intervention strategies, where targeting mechanisms underlying HPA axis hyperactivity may be effective in reducing the risk of depression in adolescence.

Another important point emerging from this review is changes in association between cortisol and depression depending on the time when cortisol was measured. No associations were found between cortisol and adolescent depression when measured diurnally, in the afternoon or in the evening. From this, it can be concluded that the time of the day cortisol is collected is important in determining whether or not there is an association between cortisol and MDD. It is clear that particular times of the day hold more relevance with regards to HPA axis activation and adolescent depression, which is not surprising considering cortisol production is dependent on circadian rhythm and peaks in the first hour after awakening (Adam et al., 2010). Specifically, awakening time, morning, and night-time appear to be important times which reveal critical associations between HPA axis activity and adolescent depression.

There are some limitations in this current review that should be noted. Firstly, heterogeneity in methodologies used in studies was high. Furthermore, it was not possible to conduct a sensitivity analysis to assess the source of heterogeneity due to the small sample size for the cortisol subgroups. This, combined with the low number of studies available, highlights the need for further research with more comparable inclusion criteria and methodology. Our findings also revealed that most studies focused on adolescent populations aged up to 19 years old, highlighting the need to conduct research combining adolescents and young adults to capture the biopsychosocial changes occurring during this transition period. A further limitation of our review is the limited number of studies conducted in LMICs. Our systematic review revealed the sparsity of research looking at how HPA axis activity is involved in adolescent depression worldwide, with only three studies conducted in LMICs. It is striking to see how little has been done in trying to understand how the HPA axis, one of the key biological systems known to be involved in the pathogenesis of MDD in adults, is involved in MDD development in adolescence, especially given that youth in LMICs comprise 90% of the world’s child and adolescent populations (Avenevoli et al., 2015, Kieling et al., 2011). Our findings further highlight the importance of conducting such research, with particular emphasis on, but not exclusive to, LMICs where adolescence population rates are the highest, bearing the greatest burden of depression (Kieling et al., 2011).

In conclusion, this is the first systematic review and meta-analysis looking at cortisol and MDD specifically in adolescents and young adults, and both longitudinally and cross-sectionally. We found evidence suggesting that elevated morning cortisol is associated with increased risk of developing adolescent depression. However, the number of studies remains low with the majority coming from HICs, underlining the importance of conducting further research to better understand the biological mechanisms underpinning depression in adolescence and young adulthood across the globe.

## Declaration of Competing Interest

Dr Mondelli has received research funding from Johnson & Johnson as part of a research program on depression and inflammation, but the research described in this paper is unrelated to this funding. All other authors declare they have no conflicts of interest to report.
